# One Health adjuvant selection for vaccines against zoonotic infections

**DOI:** 10.37349/emed.2025.1001316

**Published:** 2025-05-07

**Authors:** Anna Antipov, Nikolai Petrovsky

**Affiliations:** 1 Vaxine Pty Ltd, Warradale, Adelaide, South Australia 5046, Australia; 2 Australian Respiratory and Sleep Medicine Institute, Adelaide, South Australia 5042, Australia

**Keywords:** Vaccine, adjuvant, One Health, veterinary, human, infection, immunity

## Abstract

Vaccines are typically designed either for human or veterinary use. Using One Health principles it would be more efficient to develop a single vaccine to cover all animal and human species at threat from a specific pathogen. A major issue for designing One Health vaccines is that some commonly used human adjuvants such as aluminium salts are not suitable for some animal species, such as felines, where they can cause injection site sarcomas. Conversely, some commonly used animal adjuvants such as mineral oil emulsions are too reactogenic to be used in humans. In addition, species-specific differences in innate immune receptors such as Toll-like receptors (TLR) may mean an adjuvant that works in one species does not work in another. This review presents an overview of human and veterinary adjuvants in use and from this list identifies those that might be most suitable for use in a One Health vaccine strategy. Two notable adjuvant candidates already supported by both human and animal data are squalene oil emulsions and delta inulin-CpG combination adjuvant known as Advax-CpG55.2. These two adjuvants have already been shown to be safe and effective across multiple species including when formulated in influenza vaccines. This could be highly relevant to adjuvant selection for vaccines in development against the current North American bovine H5N1 avian influenza outbreak with the potential need to cover multiple susceptible species including birds, cattle and cats in addition to humans. Additional considerations for One Health adjuvants would be suitable administration routes and dosing across species of widely varying size, physiology and genetics. The availability of adjuvants such as squalene emulsions and Advax-CpG55.2 with broad species activity and safety, including in humans, should make One Health vaccine approaches more common in the future.

## Introduction

One Health is defined by the World Health Organisation (WHO) as “*an integrated, unifying approach to balance and optimize the health of people, animals and the environment*” [1]. This principle recognizes the critical interconnections between human and animal health and their relevance to potential global health threats [2]. In recent years, the One Health approach has gained attention in the field of vaccine development due to its potential to help tackle the emergence and spread of infectious diseases involving both animals and humans [3]. This review explores the application of One Health approaches to vaccine adjuvant selection, including addressing potential issues of species-specific effects of different adjuvant types.

One Health is both relevant to infections transmitted between animals and humans and vice versa. Notably, most new human infectious diseases are zoonotic in origin [4]. A prime example was the sudden emergence of the first human outbreak of SARS coronavirus in 2002, with the outbreak being traced to a bat virus that had crossed over to the human population via civet cats as an intermediary [5]. Many other zoonotic infection examples exist [6], including MERS coronavirus [7], Ebola [8], and avian influenza [9]. In such situations it is critical to consider not only the human disease, but also the potential animal and environmental reservoirs, so an integrative pan-species strategy of control can be implemented. Cross-species transmissions can have significant implications to public health, with factors such as changes in husbandry practices to accommodate expansion of pig, cattle and poultry production leading to environments more conductive to the emergence and spread of zoonotic infections [10, 11]. The emergence and re-emergence of infections across multiple species including avian influenza [12], Japanese encephalitis [13], and others [14], highlights the importance of having One Health vaccines available to simultaneously protect both humans and animals.

Adjuvants enhance vaccine immunogenicity leading to increased protection, but it is vital this not compromise vaccine safety [15]. In addition, adjuvants may allow for antigen-sparing, increased duration of protection and reduced need for boosters [16, 17]. The major classes of adjuvant and their advantages and disadvantages are shown in Table 1. Although vaccines used for humans and for production or companion animals have the same goal of protection, requirements may differ between these groups. Considerations for veterinary adjuvants include the costs of goods, whether the animal is for human consumption or companionship, rearing practises, herd epidemiology and any potential negative impact on animal growth or carcass blemish [18]. For human vaccines, efficacy, safety and tolerability are the top priority, with cost of goods a lower priority than when selecting adjuvants for vaccines for production animals [19].

Veterinary vaccine development is often quicker than human development due to safety and efficacy studies being able to be performed directly in the target species. However, veterinary vaccines generally have low pricing relative to human products. For example, the most successful animal vaccine which is for foot-and mouth disease has only 10–20% of the market value of the human papillomavirus vaccine [20]. Overall, the human vaccine market is 30 times the size by value of the veterinary vaccine market [21]. Human vaccines commonly cost upwards of $100 per dose, whereas livestock vaccines to be commercially viable may need to be priced at less than a $1 per dose [22].

## Adjuvants currently used in human vaccines

A summary of currently licensed human adjuvants is presented in Table 2. Since 1926, when aluminium salts were first introduced as adjuvants by Alexander Glenny, there has been limited development of new adjuvants. Until the 1990’s, only aluminium adjuvants were licensed for human use. Toxicity issues limited human use of more inflammatory adjuvants such as Freund’s complete adjuvant or other mineral oil adjuvants [23, 24]. MF59, a squalene oil emulsion adjuvant was licensed as part of an human seasonal influenza vaccine introduced in Europe in 1997. More recently, a handful of additional adjuvants have progressed to licensure in human vaccines including Advax-CpG55.2, Matrix M, CpG1018, alum-CpG1018, Alhydroxyquim AS01, AS02, and AS04 adjuvants [25]. Hence, major advances in the adjuvant field have occurred in the last two decades, with the greatest number of new human adjuvant approvals occurring only recently during the COVID-19 pandemic [26]. This opens the door for new human adjuvants to be utilised as part of a One Health vaccine strategy.

## Adjuvants used in veterinary vaccines

Some adjuvant compounds such as mineral oil emulsions are solely used in animal vaccines, due to their excess reactogenicity in humans [27]. A summary of currently licensed veterinary vaccines and adjuvants is presented in Table 3. The veterinary field is highly cost sensitive and hence generic adjuvants such as aluminium salts and oil emulsions are the most extensively used [27]. These adjuvants are over 100 years old and are non-proprietary, with multiple suppliers and a low cost of goods. On the negative side, aluminium salts have relatively weak immunogenicity and are poor inducers of cellular immunity [28]. Similarly, mineral oil emulsion adjuvants are cheap but also tend to be Th_2_ polarising [29] and are highly inflammatory causing issues such as hide scarring [30]. There are currently over 20 companies that produce adjuvanted veterinary vaccines. Domínguez-Odio et al. [10], identified that 86.9% of 351 commercial veterinary vaccines used a single adjuvant with the remaining 13.1% combining several adjuvants. Aluminium salts are the most commonly used adjuvants, being in 48.1% of veterinary vaccines followed by oil emulsions in 20.5% of vaccines. Saponins are the third most commonly used veterinary adjuvant type [31]. Selection of an appropriate veterinary adjuvant is dependent on species and the type of antigen. For example, mineral oils are favoured for inactivated or recombinant protein-based vaccines in pigs [32] but are avoided in horse vaccines due to their excess reactogenicity [33].

## Adjuvants for One Health vaccine strategies

The purpose of adjuvants is to enhance vaccine-specific immune responses as well as serve as delivery vehicles [34]. Adjuvants may be used to maximise antibody production or to specifically enhance Th_1_, Th_2_ or Th_17_ cellular immune responses [15, 35]. Currently there is no universal One Health adjuvant. Different adjuvant types are associated with advantages and disadvantages (Table 1). Adjuvants can be classified as delivery systems, antigen modifiers, immune potentiators or a combination of these [15, 20]. Many veterinary adjuvants are mixtures of surface-active compounds, microbial components and/or polymers or lipids. Understanding of adjuvant mechanisms of action remains poor, frustrated by the complexity of immune system interactions in vivo which cannot be easily teased out in vitro. In general, most adjuvants either improve antigen stability, create an antigen depot to enhance antigen uptake and presentation [17] or act as immuno-stimulators [36]. A single adjuvant may have more than one mechanism of action; for example, preservation of antigen structure and stability by adsorption of proteins to aluminium salt adjuvants together with inflammasome activation both contribute to alum’s adjuvant activity [37].

## Species limitations

Many adjuvants have species-specific effects, limiting their utility for a One Health approach. Veterinary adjuvant development has progressed more slowly than for human adjuvants [38]. Factors such as stability, ease of manufacture, cost and safety are all important considerations [39]. In recent years there has been increased focus on use of synthetic and biosynthetic materials and advanced formulation techniques to produce microparticles, combination adjuvants and derivatized polysaccharides [40–42] where delivery systems are combined with immunostimulants to develop more complex adjuvant systems [43, 44]. Genetic differences in innate immune receptors such as Toll-like receptors (TLR) can influence adjuvant potency in different species. These adjuvant challenges can make it challenging to develop a One Health vaccine. During the SARS-CoV-2 global pandemic there were spill-back infections from animals to humans resulting new viral variants such as mink-adapted SARS-CoV-2 [45]. There were also reports of many different zoo species contracting SARS-CoV-2 infections from human to animal transmission [46, 47]. This emphasized the importance of the ability to vaccinate animal species alongside humans to minimise virus transmission and evolution. This is also relevant to the current North American bovine H5N1 avian influenza outbreak that has already impacted on avian, human, bovine, and feline populations, amongst others [48]. A One Health vaccine against H5N1, safe and effective across all the relevant species could be highly beneficial.

## Types of adjuvants

### Aluminium adjuvants

Aluminium adjuvants have been in use since 1926 in both human and animal vaccines, this being long before the One Health strategy was considered. They remain the most commonly used adjuvants given their ability to boost antibody responses, reduce vaccine reactogenicity (by absorbing and slowing down the release of endotoxins), low cost, safety, stability, and ease of preparation [49]. Aluminium adjuvants are most beneficial for vaccines against extracellular pathogens given their ability to increase antibody responses via enhanced Th_2_ immunity. Despite their ubiquitous use, aluminium adjuvants have been associated with adverse side effects, some of which are species-specific. This includes the formation of large granulomas in sheep [50] and sarcomas in cats [51, 52] first noted with an alum-adjuvanted FeLV vaccine in 1991. The exact link between aluminium adjuvants and tumor formation in cats remains poorly understood but may reflect greater skin sensitivity to irritation [53]. Alum adjuvants are thereby not suitable for a global One Health strategy.

### Oil emulsion adjuvants

Oil emulsion adjuvants are generally stronger than aluminium salts and are used in large farmed species such as cattle and swine [32] as well as chickens [54] and fish [55]. Emulsion adjuvants act via induction of inflammation as well as depot formation facilitating the slow release of antigen [27]. Emulsions can be categorised as water-in-oil (WO), oil-in-water (OW) and water-in-oil-in-water (W/O/W). Cattle and poultry are most commonly vaccinated with W/O emulsions, whilst swine are vaccinated with O/W emulsions [56]. Emulsion adjuvants are associated with local and systemic reactions including fever, granulomas, abscesses, and scarring [32]. They are also quite viscous making them difficult to inject. Mineral oil emulsions may also have the risk of contamination by carcinogenic polycyclic aromatic hydrocarbons [57]. Accidental injection of human handlers with veterinary vaccines containing oil adjuvants can cause major local tissue injury due to their highly inflammatory nature [58]. This means mineral oil adjuvants are unsuitable for a One Health vaccine strategy. Only specific oil emulsion adjuvants such as those based on squalene oil are sufficiently safe and non-reactogenic for human use.

### Saponin adjuvants

Saponins are glycosides that are found in plants, fungi, and some marine animals. Saponin adjuvants increase antibody production as well as enhancing cellular immunity [31]. Human saponin adjuvants such as QS21 have haemolytic activity [59] and can have stability issues [19]. They are painful to inject although this may be reduced by formulating them into liposomes such as immune-stimulating complexes (ISCOM) [60]. Due to the need for expensive purification, QS21 costs more than generic adjuvants such as alum or oil emulsions. While potent in mammalian species including humans, saponins are less effective in non-mammalian species for reasons that are not well understood [61].

### CpG oligonucleotide adjuvants

CpGs are synthetic oligonucleotides which act as TLR ligands. Engagement of TLR receptors activate various signalling pathways leading to strong immune stimulatory activity. CpG ligands have undergone testing in many animal species and in combination with various vaccine candidates. Due to their stability and ease of synthesis, CpGs are promising molecular adjuvants that are effective in large farm animals [62, 63]. Synthetic TLR9 agonists potently activate Th_1_ immunity [64]. A challenge is that many TLR ligands are species-specific. CpG55.2 is active against a wide range of TLR9 species including mice, hamsters, ferrets, monkeys and humans [42]. Advax-CpG55.2 is a combination adjuvant where CpG55.2 is added to delta inulin to further enhance its activity and induce more potent CD8^+^ T cell responses. Advax-CpG55.2 was a key component of the SpikeVet^™^ One Health COVID-19 vaccine that was shown to be safe and effective in over 38 species from the orders Carnivora, Primates, and Artiodactyla [65]. Another CpG is CpG1018 which is used as an adjuvant in an FDA-licensed hepatitis B vaccine (Heplisav^™^) [66] and is also used in combination with alum in a human COVID-19 vaccine [67].

### Advax^®^ delta inulin adjuvant

Advax^®^ delta inulin adjuvant is a polysaccharide adjuvant that is a key component in a human COVID-19 vaccine (SpikoGen^®^) licensed in the Middle East where 8 million doses were delivered [42]. Advax^®^ alone or in combination with CpG55.2 has been shown to be safe and effective in a large array of animal species including mammals, reptiles, and birds [68]. Advax^®^ is derived from inulin, a plant-derived polysaccharide consisting of a linear fructose chain with a terminal glucose monomer. When crystallized into the microparticulate delta polymorphic form it promotes recruitment of neutrophils, macrophages and monocyte enhancing both humoral and cellular immunity. Advax^™^ particles are recognized by DC-SIGN, a human C-type lectin expressed by immature dendritic cells [69]. This helps promote antigen uptake and presentation by MHC class I and II molecules. This in turn enhances antibody production while also inducing memory CD4^+^ and CB8^+^ T cells. Advax^®^ is an ideal One Health vaccine adjuvant as it does not cause injection site inflammation that might lead to animal distress or hide scarring. Rigorous testing has shown Advax^™^ alone or in combination with CpG to be safe and effective in multiple animal species including mice, guinea pigs [70], hamsters [71], ferrets [72], rabbits [73], goats [74], macaques [75], horses [76], and camels and alpacas [77]. During the SARS-CoV-2 pandemic an Advax-CpG55.2 adjuvanted COVID-19 vaccine (SpikeVet^™^) was successfully used to immunize a widely diverse range of zoo species including large cats and non-human primates [65].

## Routes of adjuvant administration and dosing

Adjuvant use requires careful consideration of dosing and routes of administration. Currently, the most common routes of vaccine administration in most animals are subcutaneous (SC) or intramuscular (IM) although intranasal or inhaled administration are used for poultry vaccines [78]. Vaccines can also be administered orally in feed or drinking water, such as used for swine, fish and shrimp vaccination [79]. There is increasing interest in intranasal and oral vaccines due to ease of administration and induction of mucosal immunity at the point of pathogen entry [80, 81]. Potential mucosal adjuvants include bacterial toxins such as cholera toxin as well as TLR agonists such as CpG oligonucleotides [39, 82].

## Considerations for One Health vaccine use

As noted, the majority of initial vaccine studies are conducted in laboratory small-animal models meaning the translation from small to large animals is not always a straightforward task. Issues include species-specific differences in immune receptors, molecular pathways and physiology [83]. Testing of vaccines in inbred mouse strains may fail to expose issues encountered in out-bred populations [84]. For example, IL-10 shifts Th_1_-Th_2_ balance in mice but not in cattle [85]. The effects of an adjuvant in one species may not predict its effects in other species [26]. Antigen and adjuvant dosing is not necessarily proportional to body size (allometric scaling) as adjuvant effects may be independent of systemic distribution and are more dependent on local or regional distribution involving immune cells at injection sites and draining lymph nodes [86]. For example, an optimal antigen dose for a mouse was found to be one-tenth the human dose despite a 3,000-fold difference in body weight [84]. Traditional adjuvants such as oil emulsions may increase adverse effects associated with the vaccine antigen, such as fever, soreness, lethargy and autoimmune reactions [80, 87]. Depending on country and local regulations many veterinary vaccine manufacturers were not routinely required to report adverse effects or update labels post-market approval [52]. There has been a push to change this and agencies such as the US Department of Agriculture (USDA), the Canadian Department of Agriculture, and Australian Pesticides and Veterinary Medicines Authority have now made it mandatory to report adverse effects of veterinary vaccines [88].

The focus of veterinary vaccines is centred around companion and production animals, meaning the most data is available for mice, guinea pigs, rabbit, chicken, feline, monkey, sheep, pig, bovine, and equine species. Poultry, swine and ruminants (cattle, sheep and goats) account for approximately 86% of all adjuvant manufactured for veterinary vaccines, with fish, rabbits, equines and companion animals (dogs and cats) accounting for only 14% of adjuvant used [10]. Currently, knowledge of specific vaccine action is limited for most species outside of laboratory rodents, companion and farmed animals and there is a lack of data of adjuvant effects in exotic animal species. Selection of an appropriate veterinary adjuvant depends on multiple factors, such as species sensitivity, disease and type of antigen, desired immune response, and genetic differences. Current knowledge gaps make it difficult to know the optimal adjuvant regimen for exotic species. Hence, use of vaccines and adjuvants in exotic species is performed in the context of very limited knowledge and experience [89].

Veterinary vaccines are divided into core and non-core categories; with core vaccines protecting against globally distributed life-threatening diseases (e.g., rabies, distemper, feline panleukopenia), and non-core vaccines being used in specific contexts dependent on location, environment and lifestyle of the animal (e.g., *Bordetella*, Lyme disease, Feline leukemia virus). Currently most exotic species that require vaccinations have their vaccination protocols and doses extrapolated from data in domestic animals [89]. Vaccines licensed for domestic animals are commonly used off-label in exotic species. For example, carnivorous species that are susceptible to canine distemper virus such as the red panda and wolf are often given recombinant canarypox vaccines licensed for use to prevent for canine distemper virus in domestic ferrets [89, 90]. This is not ideal. Due to the time and resources required for adjuvant evaluation, there is a major divergence between what is researched and what is ultimately commercialised. Development of optimized adjuvant formulations for veterinary vaccines remains relatively under-explored [91].

## Conclusions

The One Health approach is ideal for development of vaccines to control spread of zoonotic infections across human and animal populations. Identification of optimal adjuvants for use in One Health vaccine strategies is a major priority. An ideal One Health adjuvant platform should have a low cost of goods and demonstrated safety and efficacy across humans and diverse animal species. Alongside squalene oil emulsion adjuvants, a good example of a One Health adjuvant is Advax-CpG55.2, a human vaccine adjuvant that has also been confirmed to be safe and effective across more than 40 different exotic zoo species including feline species. Availability of One Health adjuvants will assist development of vaccines to protect both human and animal populations from zoonotic diseases, with a major current focus being on development of One Health vaccines to protect against the current North American H5N1 avian influenza outbreak.

## Figures and Tables

**Table 1.** Examples of types of adjuvants, their mode of action and their key advantages and disadvantages

Adjuvant	Mode of action	Representative examples	Type of antigen	Advantages	Disadvantages
Mineral salts	Retain antigen at site of injection (short-term depot) and induce Th_2_ responses	Aluminium hydroxide Aluminium phosphate Brands: Alhydrogel, Adjuphos, Imject Alum	Extracellular pathogens Live virus Inactivated virus	Good safety profile Low cost Strong humoral response	Multiple injections often necessary High reactogenicity in felines (abscess, sarcomas, and granulomas) Adsorption based on characteristics of antigen Does not induce Th_1_ immunity Cannot be easily frozen or lyophilized
Oil emulsions	Form antigen depot at injection site and induce inflammatory cytokines	MF59 AS03 Emulsigen-D Montanide	Live virus Inactivated virus	Strong Th_2_ immunity Low cost Long term immunity	Weak Th_1_ response Scar tissue formation and adhesion Granuloma and cyst formation Inflammation, irritation and pain at injection site Reactogenicity (injection site reactions) Potential contamination from carcinogenic hydrocarbons
Immune-stimulating complexes (ISCOM)	Activate inflammasome, induce DNA release activate TLRs, induce T cell and humoral responses	Saponins Brands: Quil A, QS21, ISCOM, VetSap	Viral Bacterial Parasitic	Strong humoral and cellular immune response	Potential toxicity Haemolysis Granulomas Local inflammatory reactions Pain at injection site
Bacterial products and derivatives	Activate TLRs and elicit strong humoral and T cell responses	Monophosphoryl lipid A (MPL) Alum + MPL (AS04)	Protein Subunit	High antibody responses Mucosal or transcutaneous use	High reactogenicity (fever, arthritis, uveitis) Poor consistency between preparations Not cost-effective
Cytokines	Activate antigen presenting cells and provide co-stimulatory signals to B cells and T cells	Granulocyte-macrophage colony stimulating factor (GM-CSF)	Cancer	Good antitumor immunity	Limited application due to poor stability and toxicity High cost May promote autoimmunity
Particles (nano- and micro-)	Encapsulate antigen in biodegradable polymers, providing depot effect and targeting of antigen to antigen presenting cells	Poly(*D*,*L*-lactide-*co*-glycolic acid) polymer ester (PLGA) Poly(lactic acid) (PLA) Chitosan Polyphosphazenes	Recombinant protein DNA	Controlled release of antigen Reduced inflammatory response Biodegradable and biocompatible	Antigen release rate influenced by type of microparticle. Dosage may be difficult to optimise Antigen stability issues during production and storage
TLR ligands	Engage TLRs leading to cytokine expression and Th_1_ activity	Poly I:C CpG MPLA	Viral Bacterial Parasitic Protein	Can stimulate Th_1_ immunity and mucosal immunity Small size Good stability	High doses may result in splenomegaly Can trigger cytokine release syndrome
Polysaccharides	Stimulate both cellular and humoral immunity via DC-SIGN activation and activate complement pathway	Delta inulin (Advax^®^)	Viral Bacterial Parasitic Toxin Recombinant protein	Does not require adsorption of antigen Can be combined with other adjuvants	None identified
Combination adjuvants	Combination of immune stimulators with antigen delivery systems	Advax-CpG55.2 Alum + MPLA Alum + CpG	Viral Bacterial Parasitic Toxin Recombinant protein	Enhances both Th_1_ and Th_2_ immunity, thereby maximizing both neutralizing antibody as well as cellular immunity	See data on individual components

TLRs: Toll-like receptors

**Table 2.** Adjuvants in licensed human vaccines

Type	First licensed	Description	Adjuvant	Vaccine examples
Alum (aluminium salts)	1920	Suspension of phosphate and hydroxide salts. Adsorption of antigens forms depot effect. Activates NALP3 inflammasome	Aluminium phosphate	Diphtheria, pertussis, tetanus (e.g., Adacel) [92]
				Pneumococcus (e.g., Synflorix) [93]
				Neisseria meningitidis (e.g., Trumenba^®^) [94]
			Aluminium hydroxide	Anthrax (BioThrax^®^) [95]
				Hepatitis B (Engerix B) [96]
				Hepatitis A (e.g., Havrix^®^) [97]
				Japanese encephalitis (Ixiaro^®^) [98]
				Neisseria menigitis (e.g., Menjugate^®^) [97]
				Pneumococcus (e.g., Prevenar) [99]
			Aluminium phosphate and aluminium hydroxide	Diphtheria, pertussis, tetanus (e.g., Boostrix^®^) [100]
			Aluminium phosphate and amorphous aluminium hydroxyphosphate sulfate	Diphtheria, pertussis, tetanus (e.g., Vaxelis^®^) [101]
			Amorphous aluminium hydroxyphosphate sulfate	Human papilloma virus (e.g., Gardasil^®^) [102]
				Hepatitis B (e.g., Recombivax) [103]
Oil-in-water emulsion	1997	Stabilized squalene oil in water emulsion induces inflammatory cytokines and forms antigen depot	MF59 AS03	Influenza (e.g., Fluad^®^, Pandemrix^®^) [104]
Immune potentiator	2022	Alum + TLR7/8 agonist	Alhydroxyquim	COVID-19 (Covaxin^®^) [105]
	2004	Synthetic TLR4 ligand adsorbed to aluminum hydroxide	RC-529	Hepatitis B [106]
	2013	Naturally derived TLR4 ligand adsorbed onto *L*-tyrosine	Monophosphoryl lipid A (MPL)	Pollen allergy (Pollinex^®^) [107]
	2012	TLR9 agonist CpG oligonucleotide	ISS1018	Hepatitis B (Heplisav^®^) [66]
	2022	Increased cellular and humoral immunity	Alum-CpG1018	COVID-19 (CorbeVax^®^) [108]
Combined adjuvants	2017	Liposome co-delivering MPL and QS21	AS01_B_	Shingles (Shingrix^®^) [109]
				Malaria (Mosquirix^®^) [110]
	2005	MPL adsorbed on aluminium phosphate	AS04	Human papilloma virus (e.g., Cervarix^™^) [111]
				Hepatitis B (Fendrix^®^) [112]
	2021	Delta inulin with synthetic CpG oligonucleotide	Advax-CpG55.2^™^	COVID-19 (Spikogen^®^) [42]
Saponin complex	2021	Saponin mixed with cholesterol	Matrix-M	COVID-19 (Nuvaxovid^™^) [113]

TLR: Toll-like receptor

**Table 3.** Examples of veterinary vaccine companies and adjuvants used together with target species for each adjuvant

Company	Adjuvants used in formulations	Target species
Bioveta Ltd (Czech Republic) https://www.bioveta.eu/	Aluminium hydroxide + Quil A	Cattle
	Aluminium hydroxide	Dog, cat, cattle, sheep, goat, horse, rabbit
	Algedratum	Cattle, pig, sheep, goat, horse, camel, dog, cat, fur-bearing animals
	Algeldrat	Horse
	Oil adjuvant (Montanide ISA 35 VG)	
	Oil-in-water emulsigen	Cat
	Oil emulsion	Chicken
CEVA Sane Animale (France) https://www.ceva.com/	Aluminium hydroxide	Cattle, goat, sheep, swine
	Oil adjuvant	Cattle, buffalo
	Carbomer 971 P NF	Swine
	Oil-in-water	
Elanco (United States) https://www.elanco.com/en-us	Aluminium hydroxide	Swine
	Emulsigen D	Cattle
	Xtend III	
	Xtend SP	
Finmarkk Laboratorios S.A/Finlab (Colombia) https://finlab.com.co/	Aluminium hydroxide with low density polymers	Poultry, cattle, sheep, goat
Instituto Rosenbusch S.A (Argentina) https://rosenbusch.com/english/index.html	Aluminium hydroxide	Cattle, sheep, horse, swine, dog
BioChemiq (Argentina) https://biochemiq.com/en/	Aluminium hydroxide Gel	Horse
	Aluminium Gel and inmunomiq	
	Aluminium Gel	Cattle, goat, sheep, llama, horse
	Aluminium Gel and polymers	Horse, chicken
Kenya Veterinary Vaccines Production Institute (Kenya) https://kevevapi.or.ke/	Aluminium hydroxide and saponin	Cattle, pig, sheep, goat
	Saponin	Goat
Laborotorios HIPRA S.A (Spain) https://www.hipra.com/es	Mineral oil	Turkey, rabbit, swine, cattle, sea bass, trout, poultry, sheep, goat
	Aluminium hydroxide	Swine
Qilu Animal Health (China) https://en.qiludb.com/	Aluminium hydroxide	Cattle, sheep, camel, mink
	20% Alum Gel	Chicken, duck, goose, swine
Merck Sharp & Dohme Animal Health SL (United States) https://www.msd-animal-health.com/	*DL*-a-tocopheryl acetate	Swine
	Alum + Quil A	Ruminants
	Aluminium potassium sulphate	Cattle, sheep
Biogenesis Bago (Argentina) https://www.biogenesisbago.com/en/	Aluminium hydroxide	Canine, feline
	Oil emulsion	Cattle
CZ vaccines (Spain) https://www.czvaccines.com/en/	Alum and Quil A	Cattle, sheep
	Light mineral oil, Montanide 103, Montane 80, Polysorbate 80	Sheep, goat
	Aluminium hydroxide	Swine, canine, feline, cattle, sheep, goat
	Mineral oil (Marcol 52), Montanide 103, Montane 80	Sheep, goat, swine
	Montanide	Cattle
Vecol (Colombia) https://www.vecol.com.co/en/	Aluminium hydroxide Gel	Cattle, sheep
	Oil emulsion	Cattle, swine
Laboratorios Microsules (Uraguay) https://www.laboratoriosmicrosules.com/en/	Double emulsion adjuvant	Bovine, ovine, caprine, swine, cattle, sheep
	Montanide IMS 3012	Horse
	Montanide 3012 SEPPIC	
	Aluminium hydroxide	Bovine, camelid, equine, ovine, caprine, goat, swine, sheep, canine, feline
	Saponin	Bovine, ovine, swine, caprine
	Oil emulsion	Cattle
VetVaco (Vietnam) https://vetvaco.com.vn/en	Double oil emulsion	Swine, ruminants
	Water-in-oil or oil-in-water	
	Glycerin	Cattle, swine, horse, sheep
	Aluminium Gel	Cattle, swine, dog, cat, horse, sheep, buffalo, cow, weasel
	Skim milk	Duck, chicken, swine, dog
	Agar	Swine
Virbac (France) https://au.virbac.com/home.html	Water-in-oil-in-water emulsion	Cattle
	Aluminium	Cattle, sheep
	Alum plus saponin	Dogs
Central Region Veterinary Institute - VINODA (Vietnam) https://vinoda.vn/	Aluminium hydroxide	Swine, goat, sheep, cattle, buffalo, chicken, duck, goose, ostrich
Zoetis (United States) https://www2.zoetis.com.au/	Aluminium hydroxide + mineral oil	Cattle
	Aluminium hydroxide	Equine
	Oil adjuvant	Swine, cattle, sheep
	Aluminium phosphate + aluminium hydroxide	Cattle
Veterquimica (Chile) https://www.veterquimica.cl/	Aqueous polymer	Swine, fish (salmon and trout)
	Aluminium hydroxide	Bovine, equine
	Oil and saponin	Fish (salmon and trout)
	Oil adjuvant	
Calier (Spain) https://www.calier.com/en	Aluminium hydroxide	Avian
	Aluminium oxide	Cow, sheep, goat, pig
Vira Vaccine Shaya (Iran) https://viravaccine.com/	Aluminium hydroxide and saponin	Sheep, goat, cattle
	Oil adjuvant	
Vaxxinova (Italy) https://vaxxinova.us.com/	Oil emulsion	Poultry, fish
	Oil-in-water	Cattle
	Amplivac^™^ (formerly T56)	Swine
	Trigen^™^	
Grand Pharma (Pakistan) https://grand-pharma.com/	Aqueous gel-based adjuvant	Poultry
	Oil adjuvant Water-in-oil	
M.C.I. Sante Animale (Morocco) https://mci-santeanimale.com/	Aluminium hydroxide	Camel, cattle, sheep, goat
	Oil adjuvant	Sheep, goat, cattle
Labovet (Brazil) https://labovet.com.br/en	Saponin	Cattle, sheep, goat, donkey, pig
	Aluminium hydroxide	Cattle, buffalo, sheep, goat, horse, mule, canine, feline
Ouro Fino Saude Animal Participacoes (Brazil) https://www.ourofinosaudeanimal.com/en/	Aluminium hydroxide	Cattle, goat, horse
	Mineral oil	Cattle, buffalo
Biovac Ltd (Israel) https://biovac.co.il/	Oil adjuvant	Poultry
Vaxine Pty Ltd/Vetvax Pty Ltd (Australia) https://vaxine.net/	Advax (delta inulin)	Mice, guinea pig, hamster, ferret, rabbit, goat, monkey, horse, camel, alpaca
	Advax (delta inulin) plus CpG55.2	
		Additional 38 exotic animal species from orders Carnivora, Primates and Artiodactyla
Central Veterinary Research Laboratory (United Arab Emirates) https://www.cvrl.ae/	Alum	Horse, camel
	Advax + CpG	

## Abbreviations

TLRs: Toll-like receptors
